# Submarine channels formation driven by turbidity currents interacting with an erodible bed

**DOI:** 10.1098/rspa.2022.0137

**Published:** 2022-07

**Authors:** Rajesh K. Mahato, Subhasish Dey, Sk Zeeshan Ali

**Affiliations:** ^1^ Department of Civil Engineering, Indian Institute of Technology Kharagpur, Kharagpur 721302, West Bengal, India; ^2^ Department of Civil Engineering, Indian Institute of Technology Hyderabad, Hyderabad 502284, Telangana, India

**Keywords:** turbidity currents, instability, sediment transport

## Abstract

In this article, we explore the submarine channel formation driven by the interaction of turbidity currents with an erodible bed. The theoretical analysis considers the three-dimensional continuity and momentum equations of the fluid phase, and the advection–diffusion and Exner equations of the solid phase. The governing equations are linearized by imposing periodic perturbations on the base flow. We study the response of both the base flow (profiles of velocity and suspended sediment concentration) and perturbations (growth rate and perturbation fields) to changes in key parameters related to the flow and sediment transport. The growth rate and the critical wavenumber are examined for a given quintet formed by the gravitational parameter, longitudinal bed slope, sediment concentration at the edge of the driving layer, Rouse number and erosion coefficient. The critical wavenumber reduces with an increase in gravitational parameter, longitudinal bed slope, sediment concentration at the edge of the driving layer and erosion coefficient, while it increases with the Rouse number. For the submarine channel formation, we identify the upper threshold values for the gravitational parameter, longitudinal bed slope, sediment concentration at the edge of the driving layer and erosion coefficient and the lower threshold value for the Rouse number.

## 1. Introduction

**Figure 1.**
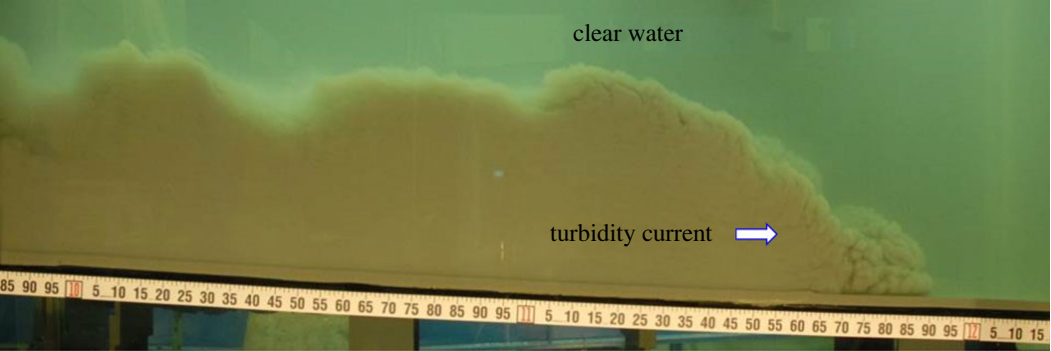
Photograph of a turbidity current in an experimental channel. (Courtesy of Octavio E. Sequeiros, Shell Global Solutions International B.V., The Netherlands.) (Online version in colour.)

Turbidity currents are ubiquitous underflows emerging in a marine environment. A photograph of a turbidity current in an experimental channel is shown in figure 1. The turbidity currents are driven by the excess density owing to the presence of suspended particles [1–4]. The origin of turbidity currents has been thought to be linked with the sediment carried by rivers, earthquakes, slope failures and tsunamis [5,7]. Analogous to rivers, turbidity currents can travel nearly hundreds to thousands of kilometres before their dissipation and deposition [8,9]. Extensive studies over the years have investigated the hydrodynamic and morphodynamic aspects of turbidity currents [7,10,11]. The subtle interaction of a turbidity current with an erodible bed helps the formation of submarine topographical features, e.g. submarine canyons, fans, levees, sediment waves, graded beddings and channels [12–15]. Among them, submarine channels are frequently observed topographic patterns on continental slopes. They act as a route of sediment transport in a submarine environment [16,17]. The flow in a submarine channel is linked with the progradation of continental slopes [16]. Therefore, an investigation of the underlying mechanism of the submarine channel formation is an important aspect.

A few studies have explored the process of flow stripping, migration and flow deposit in submarine channels [18–20]. However, a handful of attempts have been made to gain an in-depth understanding of their formation process [6,21]. Field observations have documented the existence of nearly parallel submarine channels [22], which are generated by the fundamental instability mechanism [21]. In this context, it is worth highlighting that the instability analysis is a powerful analytical tool, which has been well documented to retrace the origin and formation of various rhythmic patterns in fluvial and marine environments [21,23–26]. The instability analysis of channel inception started with the seminal work of Smith and Bretherton [27]. They found that under suitable conditions, small amplitude perturbations lead to the development of terrestrial channels. Reanalyzing the problem of terrestrial channel initiation, Loewenherz [28] discovered that during the instability process, the advection of eroded sediment dominates the diffusion. Incorporating a threshold condition for a flatbed erosion, Izumi and Parker [29] performed a linear stability analysis to investigate the terrestrial channel formation. Their formulation predicted the characteristic wavelength of terrestrial channels. In addition, they discovered that the channelization is initially triggered far down slope, and thereupon the channel head migrates upstream through the head cutting. Revelli and Ridolfi [30] revisited the analysis of Izumi and Parker [29] considering a non-flatbed. They observed that the consideration of bed curvature significantly alters the characteristic wavelength of terrestrial channels. In addition, by means of a linear stability analysis, Izumi and Parker [31] explored the process of terrestrial channel inception on hill slopes with a smooth downward-concave profile. They found that the transverse perturbations grow in time, reflecting the inception of channelization. They also predicted the characteristic wavelength associated with the maximum growth rate of perturbations.

Attempts have been made to channelize a submarine environment. Izumi [6] performed a linear stability analysis to examine the submarine gully formation due to turbidity currents. The theoretical investigation yielded a range of characteristic wavelengths for submarine gullies, being consistent with the field observations. The aforementioned studies on the terrestrial or submarine channel formation employed the depth-averaged equations of fluid phase. Hence, they could not provide an insight into the near-bed flow structure. On that account, Hall *et al*. [21] performed a linear stability analysis employing the three-dimensional equations of the fluid phase. They modelled the turbulent diffusivity as a constant throughout the flow layer. They analyzed the two-way feedback between the transverse flow structure and the sediment concentration in the form of counter-rotating longitudinal vortices. They found that for the initiation of instability, the sediment concentration of the base flow in the vertical direction must decay slowly compared to the decay of shear stress.

The brief literature survey suggests that the mechanics of the submarine channel formation needs to be explored more thoroughly. In this study, we perform a linear stability analysis, as an advancement of the study by Hall *et al.* [21], to explore the formation of parallel and uniformly spaced submarine channels driven by turbidity currents. We employ the three-dimensional flow equations driven by the suspended sediment, the advection–diffusion equation of the suspended sediment concentration and the Exner equation of sediment continuity. Unlike the conventional analysis, we use a parabolic profile of the turbulent diffusivity to capture the near-bed flow field. The behaviours of the base flow (e.g. profiles of velocity and suspended sediment concentration) and the perturbations (growth rate and perturbation fields) are explored in detail. The sensitivity of the physical system to changes in key parameters is also examined.

This article is organized as follows. In §2, the governing equations are presented. The linear stability analysis is performed in §3. The computational results are discussed in §4. Finally, the conclusion is drawn in §5.

## 2. Governing equations

**Figure 2.**
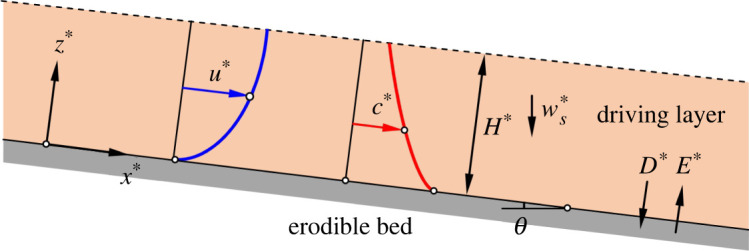
Schematic of the physical system. The broken line shows the edge of the driving layer. (Online version in colour.)

The flow field in a turbidity current can be divided into two layers: (i) the lower ‘driving layer’, which extends up to the level of maximum longitudinal velocity, and (ii) the upper ‘driven layer’, which exists above the driving layer. Luchi *et al.* [9] reported that the amount of suspended sediment in the driving layer is larger than that in the driven layer. The flow in the driving layer remains almost independent of that in the driven layer [9]. In addition, the driving layer can continue endlessly at a steady state [9]. With these facts, we consider that the flow in the driving layer triggers the instability, whereas the flow in the driven layer does not contribute to the instability mechanism. The schematic of a turbidity current of driving layer thickness $H^{*}$ over an erodible bed making an angle θ with the horizontal is shown in figure 2. A Cartesian coordinate system ($x^{*}$, $y^{*}$, $z^{*}$) is used, where $x^{*}$, $y^{*}$ and $z^{*}$ are the longitudinal, lateral and vertical distances, respectively. Hereafter, a variable with a superscript ‘asterisk’ denotes a dimensional quantity. In figure 2, $E^{*}$ and $D^{*}$ are the entrainment and deposition fluxes, respectively, and $w_{s}^{*}$ is the terminal fall velocity of sediment particles. In addition, the $u^{*}(z^{*})$ and $c^{*}(z^{*})$ represent the profiles of longitudinal velocity and sediment concentration, respectively.

Turbidity currents have been observed to be driven by small density differences (figure 1). Hence, we consider a dilute suspension of monodisperse particles with volume fraction smaller than 0.01. The volumetric displacement effects due to the particle loading are neglected. Thus, the velocity field remains divergence free. Moreover, owing to the small volume fraction of particles, the interaction between particles is trivial. In addition, we consider the particle velocity to be the sum of the local flow velocity and the terminal fall velocity. This assumption indicates that the particle velocity field is also divergence free, i.e. the particles do not accumulate in the flow field. The flow is purely driven by gravity acting on the sediment particles. For such a flow with small density differences, the use of the Boussinesq approximation is legitimate [11]. Excluding the body force terms, the Boussinesq approximation allows us to consider the mass density as a constant in the momentum equations. To proceed further, we introduce the following dimensionless variables:

$$\begin{array}{rl} & \;(x,y,z)=\frac{\left(x^{*},y^{*},z^{*}\right)}{H^{*}},\;(u,v,w)=\frac{\left(u^{*},v^{*},w^{*}\right)}{u_{f}^{*}},\\ \mathrm{and}\; & t=\frac{t^{*}u_{f}^{*}}{H^{*}},\;p=\frac{p^{*}}{\rho_{f}u_{f}^{*2}},\;\upsilon_{t}=\frac{\upsilon_{t}^{*}}{u_{f}^{*}H^{*}},\;c=\frac{c^{*}}{C_{a}}, \end{array}\}$$

where ($u^{*}$, $v^{*}$, $w^{*}$) are the velocity components in ($x^{*}$, $y^{*}$, $z^{*}$), respectively; $u_{f}^{*}$ is the shear velocity; $t^{*}$ is the time; $p^{*}$ is the pressure; $\rho_{f}$ is the mass density of fluid; $\upsilon_{t}^{*}$ is the turbulent diffusivity; $c^{*}$ is the volumetric sediment concentration; and $C_{a}$ is the reference sediment concentration. Note that the present formulation is solely focused on the fully developed turbidity currents. Applying the Boussinesq approximation, the three-dimensional continuity and momentum equations for the fluid phase are as follows [21,23]:

2.1 $$\frac{\partial u_{i}}{\partial x_{i}}=0$$

and

2.2 $$\frac{\partial u_{i}}{\partial t}+u_{j}\frac{\partial u_{i}}{\partial x_{j}}=-\frac{\partial p}{\partial x_{i}}+\frac{\partial }{\partial x_{j}}\left(\upsilon_{t}\frac{\partial u_{i}}{\partial x_{j}}\right)+\frac{\partial \upsilon_{t}}{\partial z}\cdot \frac{\partial w}{\partial x_{i}}+GSc\delta_{i1}-Gc\delta_{i3},$$

where G is the gravitational parameter, S is the longitudinal bed slope (=tan⁡θ) and $\delta_{ij}$ represents the Kronecker delta function. The gravitational parameter G, which indicates the ratio of gravity force to inertia, is expressed as follows:

2.3 $$G=\frac{\Delta C_{a}gH^{*}}{u_{f}^{*2}},$$

where Δ is submerged relative density $\left[=(\rho_{s}-\rho_{f})/\rho_{f}\right]$, $\rho_{s}$ is the mass density of sediment particles and g is the acceleration due to gravity. It is worth mentioning that the gravitational parameter G carries the same physical meaning as that of the bulk Richardson number Ri. In addition, the gravitational parameter G (or the bulk Richardson number Ri) can be related to the densimetric Froude number Fr as G (or Ri) $\propto 1/Fr^{2}$. With the assumption of insignificant particle inertia and volume fraction, the solid phase can be mathematically described by the advection–diffusion equation of suspended sediment motion as follows [32]:

2.4 $$\frac{\partial c}{\partial t}+(u_{i}-\beta \kappa Z\delta_{i3})\frac{\partial c}{\partial x_{i}}=\frac{\partial }{\partial x_{i}}\left(\upsilon_{s}\frac{\partial c}{\partial x_{i}}\right),$$

where β is the proportionality factor, κ is the von Kármán coefficient (=0.41), Z is the Rouse number $\left[=w_{s}^{*}/(\beta \kappa u_{f}^{*}\right)$], which characterizes the influence of the upward turbulent diffusion on the terminal fall velocity of particles, and $\upsilon_{s}$ is the dimensionless sediment diffusivity. The proportionality factor β depends on the centrifugal acceleration induced on the particles [32]. The determination of β is a difficult proposition. For simplicity, we consider the sediment diffusivity to be identical with the turbulent diffusivity, i.e. β=1. To proceed further, the vertical profile of turbulent diffusivity is required. Herein, we employ a parabolic profile of turbulent diffusivity, which is expressed in a dimensionless form in the following [32]:

2.5 $$\upsilon_{t}=\kappa z(1-z).$$

The parabolic profile can represent a realistic approximation for the turbulent diffusivity distribution in turbidity currents compared to a constant turbulent diffusivity assumption. The present formulation considers the suspended load as the dominant mode of sediment transport. Hence, the evolution of the erodible bed can be modelled by balancing the entrainment and deposition fluxes as follows [33]:

2.6 $$\frac{\partial \eta }{\partial t}=D-E,$$

where η is the bed elevation with respect to the base flow. We estimate the dimensionless sediment deposition flux as follows:

2.7 $$D=\beta \kappa ZC_{a}{c|}_{z=\eta }.$$

For the sediment entrainment flux, several nonlinear relations are available in the literature. However, incorporating the nonlinear relations in the linear stability analysis may not be feasible. Herein, the sediment entrainment flux E is expressed as a linear function of velocity gradient [21,23]. In the dimensionless form, it is

2.8 $$E=N{\frac{\partial u}{\partial z}|}_{z=\eta },$$

where N is the erosion coefficient ($=\beta_{e}\upsilon_{tr}^{*}\rho_{f}/H^{*}$), $\beta_{e}$ is a proportionality constant and $\upsilon_{tr}^{*}$ is the turbulent diffusivity at the reference level (z=0.01). The proportionality constant $\beta_{e}$ characterizes the sediment entrainment flux per unit area and shear stress [21,23]. The aforementioned coupled system of governing equations needs to be supplemented by the appropriate boundary conditions at the interface between turbidity current and erodible bed and at the edge of the driving layer. At the interface, we impose the no-slip condition for the longitudinal and lateral velocity components, whereas the vertical velocity component equals the rate at which the erodible bed evolves. The sediment concentration at the interface equals the reference sediment concentration. Moreover, at the edge of the driving layer, we consider a finite sediment concentration, denoted by $c_{h}^{*}$, and a vanishing longitudinal velocity gradient. The boundary conditions in a dimensionless form are expressed as follows:

2.9 $${u|}_{z=\eta }=0,\;{v|}_{z=\eta }=0,\;{c|}_{z=\eta }=1,\;{c|}_{z=1}=c_{h},\;{\frac{\partial u}{\partial z}|}_{z=1}=0\;\mathrm{and}\;{w|}_{z=\eta }=\frac{\partial \eta }{\partial t}.$$

## 3. Linear stability analysis

To study the stability of the considered physical system, we decompose the variables as follows:

3.1 $$(u,v,w,c,p,\eta )=(u_{0},0,0,c_{0},p_{0},0)+(u_{1},v_{1},w_{1},c_{1},p_{1},\eta_{1}),$$

where subscripts ‘0’ and ‘1’ denote the base flow and perturbations, respectively. The perturbations, which are expanded in the form of normal modes, are expressed as follows [21]:

3.2 $$\begin{array}{rl} & \;(u_{1},w_{1},c_{1},p_{1},\eta_{1})=[U(z),W(z),C(z),P(z),K]\sin(ky)\exp(\Omega t)\\ \mathrm{and}\; & v_{1}=V(z)\cos(ky)\exp(\Omega t), \end{array}\}$$

where U, V, W, C, P and K are the perturbation eigenfunctions, k is the dimensionless wavenumber in the lateral direction and Ω is the growth rate of perturbations. It is apparent that the perturbations are periodic in lateral direction, growing exponentially with time. The present formulation pays attention to the instability of straight longitudinal channels. Like the subaerial channels, submarine channels also manifest meandering in their course. It is worth mentioning that the instability theory can be applied to gain insights into the cause of meandering [34–36]. However, this study does not focus on this aspect. Hence, the perturbations do not evolve in the longitudinal direction. It has been observed that in turbidity currents, the flow and the sediment concentration fields attain a steady state after a certain time [9]. Hence, to continue further, we consider a unidirectional fully developed, quasi-steady base flow, where the variables remain independent of time, and longitudinal and lateral directions. With the aforementioned assumptions, equations (2.2) and (2.4) are simplified to obtain the vertical profiles of longitudinal velocity and sediment concentration as follows:

3.3 $$\frac{d}{dz}\left(\upsilon_{t}\frac{du_{0}}{dz}\right)+GSc_{0}=0$$

and

3.4 $$\frac{d}{dz}\left(\upsilon_{s}\frac{dc_{0}}{dz}\right)+\beta \kappa Z\frac{dc_{0}}{dz}=0.$$

The aforementioned equations are solved numerically with the following boundary conditions:

3.5 $$u_{0}{|}_{z=0.01}=0,\;{c_{0}|}_{z=0.01}=1,\;{c_{0}|}_{z=0.995}=c_{h}\;\mathrm{and}\;{\frac{du_{0}}{dz}|}_{z=0.995}=0.$$

In this study, the interface between the turbidity current and the erodible bed is set at z=0.01, while the edge of the driving layer is considered at z=0.995, to avoid the singularity at z=0 and z=1. At the base flow, the entrainment and deposition fluxes remain balanced. Consequently, we obtain

3.6 $$C_{a}=\frac{N}{\kappa Z}\cdot {\frac{du_{0}}{dz}|}_{z=0.01}.$$

Substituting equations (3.1) and (3.2) into equations (2.1), (2.2), (2.4) and (2.6), and after applying linearization, we obtain the following linear perturbation equations for the eigenfunctions:

3.7 $$\begin{array}{rl} & \;-kV+\frac{dW}{dz}=0, \end{array}$$

3.8 $$\begin{array}{rl} & \kappa z(1-z)\left(-k^{2}U+\frac{d^{2}U}{dz^{2}}\right)+\kappa (1-2z)\frac{dU}{dz}-W\frac{du_{0}}{dz}+GSC=\Omega U, \end{array}$$

3.9 $$\begin{array}{rl} & \kappa z(1-z)\left(-k^{2}V+\frac{d^{2}V}{dz^{2}}\right)+\kappa (1-2z)\frac{dV}{dz}+\kappa (1-2z)kW-kP=\Omega V, \end{array}$$

3.10 $$\begin{array}{rl} & \kappa z(1-z)\left(-k^{2}W+\frac{d^{2}W}{dz^{2}}\right)+2\kappa (1-2z)\frac{dW}{dz}-\frac{dP}{dz}-GC=\Omega W, \end{array}$$

3.11 $$\begin{array}{rl} & \kappa z(1-z)\left(-k^{2}C+\frac{d^{2}C}{dz^{2}}\right)+\kappa (1-2z)\frac{dC}{dz}-W\frac{dc_{0}}{dz}+\kappa Z\frac{dC}{dz}=\Omega C, \end{array}$$

3.12 $$\begin{array}{rl} \mathrm{and}\; & \kappa ZC_{a}{C|}_{z=0.01}-N{\frac{dU}{dz}|}_{z=0.01}+\left(\kappa ZC_{a}{\frac{dc_{0}}{dz}|}_{z=0.01}-N{\frac{d^{2}u_{0}}{dz^{2}}|}_{z=0.01}\right)K=\Omega K. \end{array}$$

The aforementioned equations are associated with the following boundary conditions:

3.13 $$\begin{array}{rl} & {U|}_{z=0.01}+K{\frac{du_{0}}{dz}|}_{z=0.01}=0,\;{V|}_{z=0.01}=0,\\ & {W|}_{z=0.01}=\Omega K,\;{C|}_{z=0.01}+K{\frac{dc_{0}}{dz}|}_{z=0.01}=0,\\ \mathrm{and}\; & {U|}_{z=0.995}={V|}_{z=0.995}={W|}_{z=0.995}={C|}_{z=0.995}=0. \end{array}\}$$

The domain in the vertical direction is discretized by employing the Chebyshev points as $z_{1j}=\cos(j\pi /n)$, where n is the number of grids and j ranges from 0 to n [37]. The derivatives are approximated using the Chebyshev differentiation matrix [37]. To employ the Chebyshev collocation method, the vertical domain z∈(0,1) is mapped onto $z_{1}\in (-1,1)$. The discretized system of the equations of perturbations together with the boundary conditions constitute a generalized eigenvalue problem, which is solved using the MATLAB R2021b routine *eigs*. We observed that the results become insensitive to n when n exceeds 50. Hence, for the numerical computations, we consider n=70.

For numerical experiments, it is useful to estimate the typical ranges of the key parameters. The experimental measurements of Altinakar *et al.* [38] and Nourmohammadi *et al.* [39] suggested that $G\leq O(10^{2})$. The longitudinal slope S of the natural submarine channels ranges from 0.1 to 0.001 [40]. As the major fraction of the suspended sediment belongs to the driving layer, we assume that the dimensionless sediment concentration at the edge of the driving layer follows $c_{h}\leq O(10^{-1})$. Regarding the Rouse number Z, extensive experimental studies indicated Z≤O(1) [32]. However, it is rather difficult to set a representative magnitude of the proportionality constant $\beta_{e}$. Hence, following Hall *et al.* [21], we consider the erosion coefficient to be $N\leq O(10^{-5})$, which offers an estimation of $\beta_{e}$.

## 4. Results and discussion

The mathematical formulation indicates that the instability depends on several key parameters, e.g. the gravitational parameter G, longitudinal bed slope S, sediment concentration at the edge of the driving layer $c_{h}$, Rouse number Z and erosion coefficient N.

### (a) Profiles of base velocity and suspended sediment concentration

**Figure 3.**
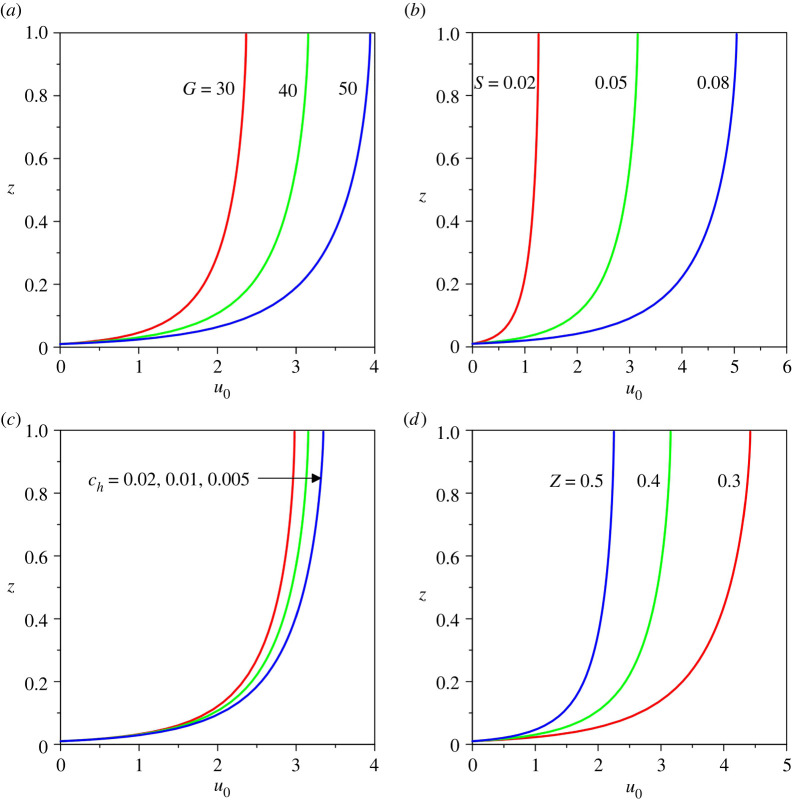
Base velocity profiles $u_{0}(z)$ for different values of (*a*) gravitational parameter G, (*b*) longitudinal bed slope S, (*c*) sediment concentration at the edge of the driving layer $c_{h}$ and (*d*) Rouse number Z. (Online version in colour.)

First, we examine the sensitivity of the base velocity and suspended sediment concentration profiles to the relevant physical parameters. To this end, figure 3*a*–*d* displays the base velocity profiles $u_{0}(z)$ for different values of gravitational parameter G, longitudinal bed slope S, sediment concentration at the edge of the driving layer $c_{h}$ and Rouse number Z, respectively. In the numerical experiment, it is found that the base velocity remains insensitive to the erosion coefficient N. At a given vertical distance z, the base velocity increases with an increase in G, S and $c_{h}$, whereas it reduces with Z (figure 3).

**Figure 4.**
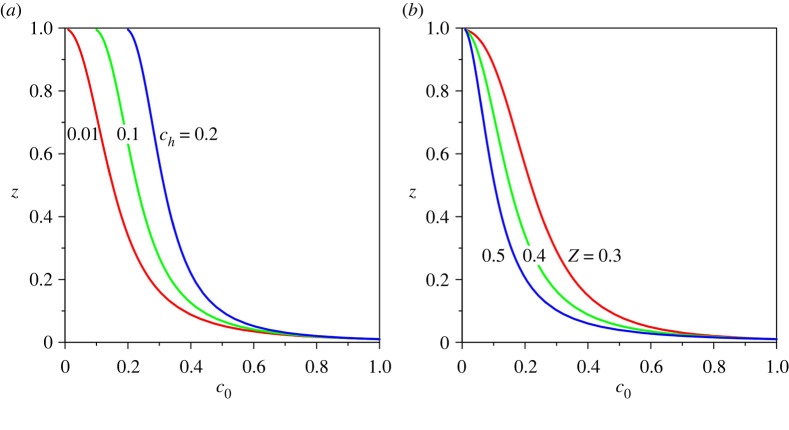
Base suspended sediment concentration profiles $c_{0}(z)$ for different values of (*a*) sediment concentration at the edge of the driving layer $c_{h}$ and (*b*) Rouse number Z. (Online version in colour.)

The base sediment concentration profiles $c_{0}(z)$ for different values of sediment concentration at the edge of the driving layer $c_{h}$ and Rouse number Z are shown in figure 4*a*,*b*. It is found that the base concentration profiles are insensitive to the gravitational parameter G, longitudinal bed slope S and erosion coefficient N. Figure 4 shows that at a given vertical distance z, the base concentration increases with an increase in $c_{h}$ but reduces with Z. At a given vertical distance, an increase in base concentration enhances the base velocity (figures 3*c* and 4*a*). This is accredited to the fact that the turbidity currents are driven by the gravitational force acting on the suspended sediment.

**Figure 5.**
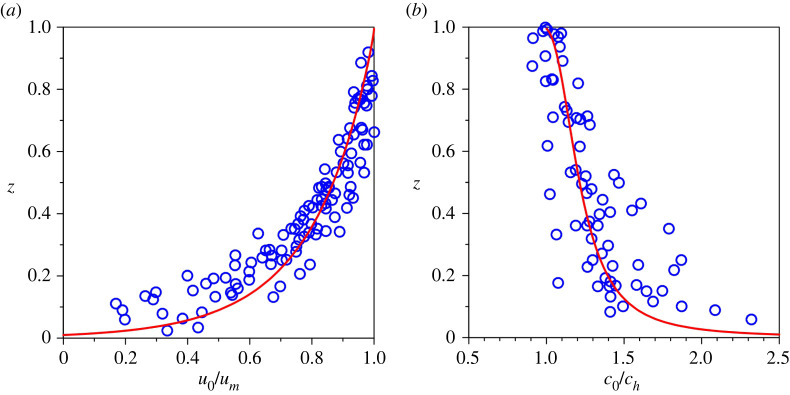
Comparison of the profiles of (*a*) base velocity and (*b*) sediment concentration with the experimental data. The gravitational parameter G=30, longitudinal bed slope S=0.05, sediment concentration at the edge of the driving layer $c_{h}=0.4$ and Rouse number Z=0.4 are considered. (Online version in colour.)

Figure 5 offers a comparison of the computed base velocity and sediment concentration profiles obtained from this study with the experimental data of Altinakar *et al.* [38], Nourmohammadi *et al.* [39] and Sequeiros *et al.* [41]. The pertinent physical parameters are kept constant depending on the experimental conditions. In figure 5, the velocity and concentration are rescaled with their respective values at the edge of the driving layer. The computed profiles of velocity and concentration agree satisfactorily with their respective experimental profiles. This observation justifies the assumption of the parabolic profile of turbulent diffusivity.

### (b) Growth rate

**Figure 6.**
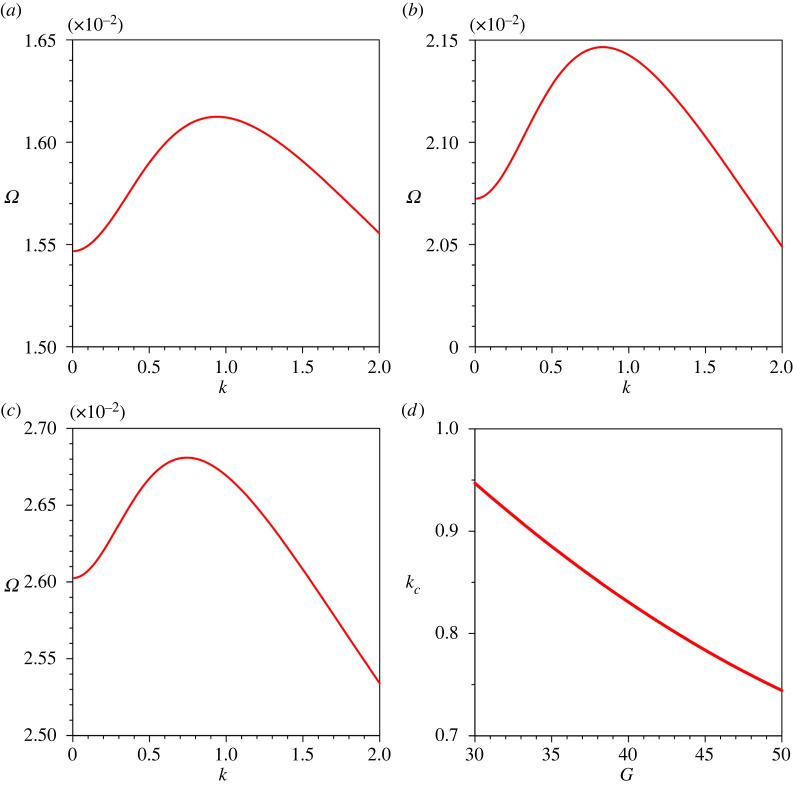
Growth rate of perturbations Ω versus dimensionless wavenumber k for different gravitational parameters G: (*a*) G=30, (*b*) G=40, (*c*) G=50 and (*d*) dimensionless critical wavenumber $k_{c}$ versus gravitational parameter G. (Online version in colour.)

**Figure 7.**
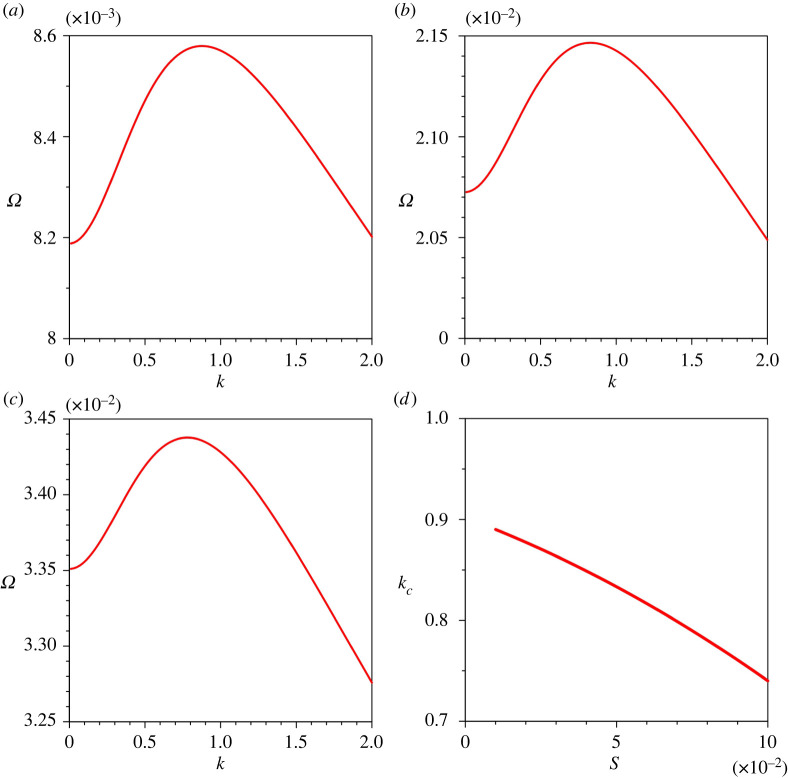
Growth rate of perturbations Ω versus dimensionless wavenumber k for different longitudinal bed slopes S: (*a*) S=0.02, (*b*) S=0.05, (*c*) S=0.08 and (*d*) dimensionless critical wavenumber $k_{c}$ versus longitudinal bed slope S. (Online version in colour.)

**Figure 8.**
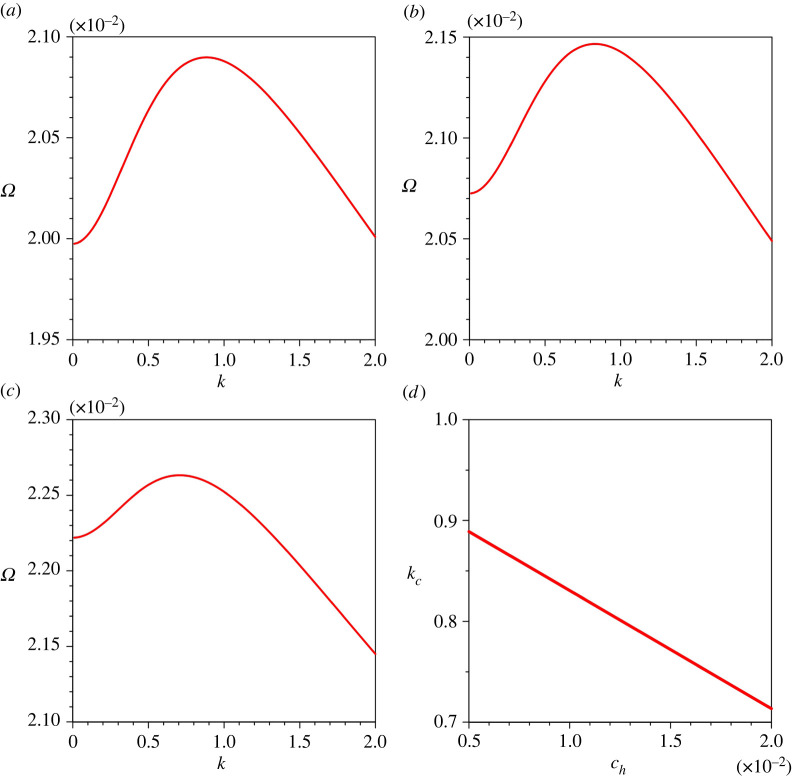
Growth rate of perturbations Ω versus dimensionless wavenumber k for different sediment concentrations at the edge of the driving layer $c_{h}$: (*a*) $c_{h}=0.005$, (*b*) $c_{h}=0.01$, (*c*) $c_{h}=0.02$ and (*d*) dimensionless critical wavenumber $k_{c}$ versus sediment concentrations at the edge of the driving layer $c_{h}$. (Online version in colour.)

**Figure 9.**
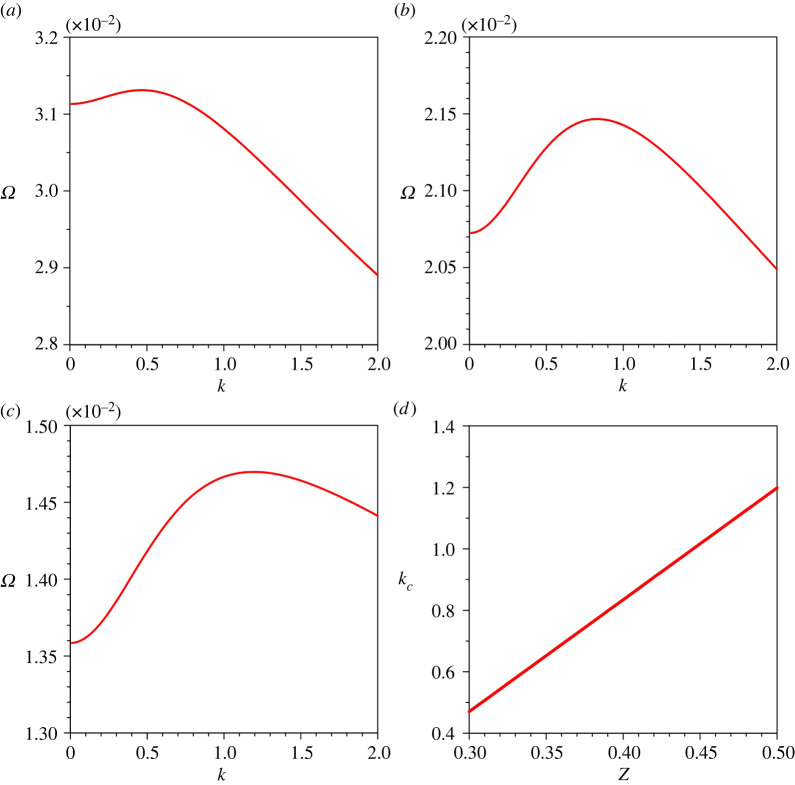
Growth rate of perturbations Ω versus dimensionless wavenumber k for different Rouse numbers Z: (*a*) Z=0.3, (*b*) Z=0.4, (*c*) Z=0.5 and (*d*) dimensionless critical wavenumber $k_{c}$ versus Rouse number Z. (Online version in colour.)

**Figure 10.**
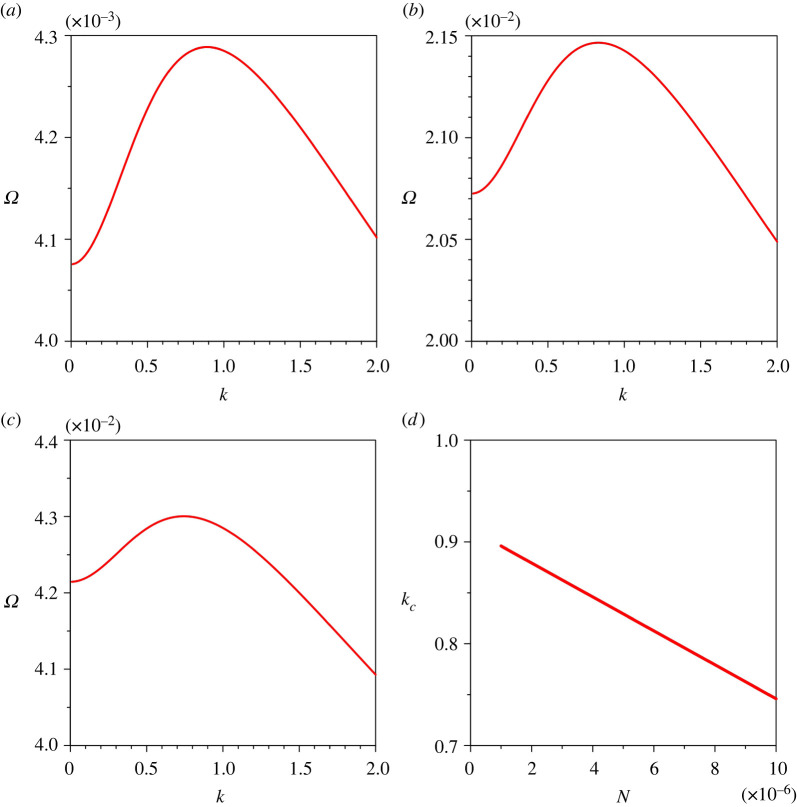
Growth rate of perturbations Ω versus dimensionless wavenumber k for different erosion coefficients N: (*a*) $N=10^{-6}$, (*b*) $N=5\times 10^{-6}$, (*c*) $N=10^{-5}$ and (*d*) dimensionless critical lateral wavenumber $k_{c}$ versus erosion coefficient N. (Online version in colour.)

We now explore the growth rate of perturbations in the parameter space. Figure 6*a*–*c* depicts the variations of the growth rate of perturbations Ω with the dimensionless wavenumber k for different gravitational parameters G. A positive (or negative) growth rate reflects the growth (or decay) of perturbations. To prepare figures 6–10, the gravitational parameter G=40, longitudinal bed slope S=0.05, sediment concentration at the edge of the driving layer $c_{h}=0.01$, Rouse number Z=0.4 and erosion coefficient $N=5\times 10^{-6}$ are taken as reference values. For a given combination of parameters, Ω amplifies with an increase in k attaining its peak value and then it follows a monotonically decreasing trend with k (see figures 6–10). For a given k, an increase in G results in an amplification of Ω (the Ω associated with $k_{c}$ amplifies from 0.0161 to 0.0268 as the G increases from 30 to 50). Hence, G plays a destabilizing role. This observation is due to the fact that an increase in G is to amplify the near-bed velocity gradient (figure 3*a*), which in turn enhances the sediment entrainment flux (see equation (2.8)). We denote the wavenumber with the maximum growth rate, called the critical wavenumber, by $k_{c}$. Figure 6*d* shows the variation of $k_{c}$ with G. The $k_{c}$ reduces with an increase in G. Therefore, an increase in sediment entrainment flux tends to form channels having the longer wavelengths.

Figure 7*a*–*c* presents the variations of the growth rate Ω with the dimensionless wavenumber k for different longitudinal bed slopes S. For a given k, Ω is amplified as the S increases owing to the enhanced sediment entrainment rate (the Ω at $k_{c}$ amplifies from 0.0086 to 0.0344 as the S increases from 0.02 to 0.08). Figure 7*d* shows the variation of the dimensionless critical wavenumber $k_{c}$ with S. The $k_{c}$ reduces with an increase in S. Hence, an increase in S favours the channel formation with longer wavelengths.

The sensitivity of the growth rate of perturbations Ω to sediment concentration at the edge of the driving layer $c_{h}$ is shown in figure 8*a*–*c*. For a given k, the Ω enhances as the $c_{h}$ increases (the Ω associated with $k_{c}$ amplifies from 0.0209 to 0.0226 as the $c_{h}$ increases from 0.005 to 0.02). This observation is attributed to the higher sediment entrainment rate owing to an increase in near-bed velocity gradient. Figure 8*d* shows the variation of the dimensionless critical wavenumber $k_{c}$ with $c_{h}$. The $k_{c}$ diminishes with an increase in $c_{h}$.

Figure 9*a*–*c* displays the variations of the growth rate of perturbations Ω with the dimensionless wavenumber k for different Rouse numbers Z. For a given k, an increase in Z suppresses the growth rate (Ω at $k_{c}$ diminishes from 0.0313 to 0.0147 as the Z increases from 0.4 to 0.6). This is due to the fact that an increase in Z enhances the sediment deposition flux (see equation (2.7)). The variation of the dimensionless critical wavenumber $k_{c}$ with Z is shown in figure 9*d*, where the $k_{c}$ increases with Z.

Figure 10*a*–*c* shows the variations of the growth rate of perturbations Ω with the dimensionless wavenumber k for different erosion coefficients N. For a given k, an increase in N is to enhance the sediment entrainment flux (the Ω associated with $k_{c}$ amplifies from 0.0043 to 0.043 as the N increases from $10^{-6}$ to $10^{-5}$). This strengthens the growth rate (figure 10*a*–*c*). The variation of the dimensionless critical wavenumber $k_{c}$ with N is shown in figure 10*d*. The $k_{c}$ reduces as the N increases.

The parametric study on the growth rate of perturbations reveals that the growth rate Ω amplifies with an increase in gravitational parameter G, longitudinal bed slope S, sediment concentration at the edge of the driving layer $c_{h}$ and erosion coefficient N, whereas it diminishes with an increase in Rouse number Z.

**Figure 11.**
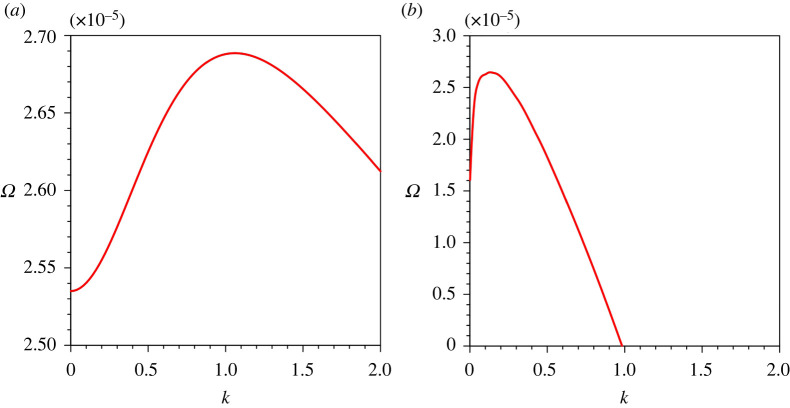
Growth rate of perturbations Ω versus dimensionless wavenumber k: (*a*) the present study and (*b*) Hall *et al.* [21]. (Online version in colour.)

Figure 11 offers a comparison of the growth rates obtained from the present study (figure 11*a*) with those obtained from Hall *et al.* [21] (figure 11*b*). For the computation, we consider G=25, $S=5\times 10^{-5}$, $c_{h}=0.01$, Z=0.4 and $N=10^{-5}$. Hall *et al.* [21] overlooked the influence of the longitudinal bed slope. It is evident that the growth rate curves follow the similar trend. In addition, both the curves display a similar magnitude of the maximum growth rate. However, in this study, the maximum growth rate appears at k=1.051 (figure 11*a*), whereas Hall *et al.* [21] observed the maximum growth rate at k=0.25 (figure 11*b*). This difference is attributed to the different length scales used for making the wavenumber dimensionless. For the length scale, the present formulation uses the driving layer thickness, whereas Hall *et al.* [21] used the ratio of the sediment diffusivity to the terminal fall velocity. Unlike the study by Hall *et al.* [21], we found that the critical wavenumber $k_{c}$ is sensitive to the key physical parameters.

It is worth discussing the predicted channel wavelength for different physical parameters. The instability mechanism yields a wide range of lateral wavelength for submarine channels. We observe that the higher magnitudes of G, S, $c_{h}$ and N, and a lower magnitude of Z predict the submarine channels having an infinitely large lateral wavelength. Intuitively, a submarine channel having a longer lateral wavelength can be deemed to be a plane bed. Hence, for the submarine channel formation having a finite wavelength, this study sets the upper threshold values for G, S, $c_{h}$ and N, and a lower threshold value for Z, as presented in table 1. In other words, the submarine channel formation having a finite wavelength is possible if the G, S, $c_{h}$ and N remain smaller than their respective upper threshold values. Similarly, the submarine channels having a finite wavelength are formed if the Z exceeds its lower threshold value. The appearance of the upper and lower threshold suggests that the lateral wavelength of the submarine channels can be of the order of kilometres. However, for a turbidity current with a driving layer thickness of *O*(10 m), the numerical experiments produce the submarine channels with a minimum lateral wavelength of *O*(70 m). The theoretical predictions agree well with the field observations, which suggest that the lateral wavelength of the submarine channels ranges from a few hundred metres to a few kilometres [42,43]. The instability analyses of Hall *et al.* [21] and Izumi [6] predicted the range of lateral wavelength as 250–2500 m and 150–8000 m, respectively. It is pertinent to mention that the parallel channels on hill slopes also exhibit a range of lateral wavelength [31]. We observe that the channel wavelength amplifies with an increase in gravitational parameter G, longitudinal bed slope S, sediment concentration at the edge of the driving layer $c_{h}$ and erosion coefficient N. However, it reduces as the Rouse number Z increases.

**Table 1.** Upper and lower thresholds of key parameters.

parameter	G	S	$c_{h}$	Z	*N*
upper threshold	170	0.26	0.04	—	$2.6\times 10^{-5}$
lower threshold	—	—	—	0.25	—

It is interesting to present the favourable condition for the submarine channel formation in terms of several dimensional variables. To this end, we consider a turbidity current with a driving layer thickness of 10 m. The upper threshold of the gravitational parameter G gives the shear velocity as $u_{f}^{*}>0.0762\;\text{m}\;{\text{s}}^{-1}$. The upper threshold of the sediment concentration at the edge of the driving layer $c_{h}$ suggests the sediment concentration to be smaller than 0.08%. The lower threshold of the Rouse number Z produces $w_{s}^{*}>7.81\times 10^{-3}\;\text{m}\;{\text{s}}^{-1}$. In addition, the upper threshold of the erosion coefficient makes the proportionality factor $\beta_{e}<6.4\times 10^{-5}$.

**Figure 12.**
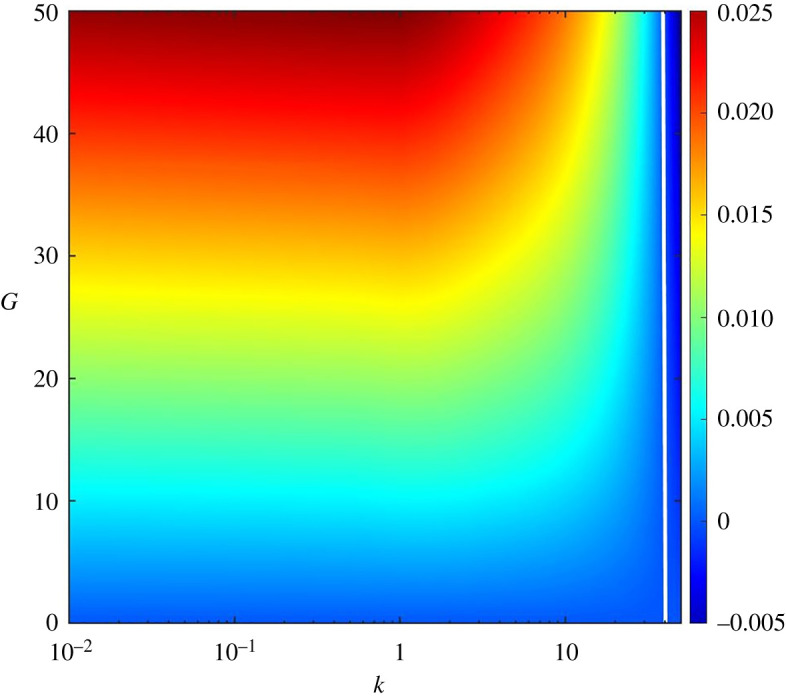
Stability diagram: gravitational parameter G versus dimensionless wavenumber k. (Online version in colour.)

It is also interesting to explore the stability diagram. Figure 12 illustrates the stability diagram on the G-k plane (i.e. gravitational parameter versus dimensionless wavenumber plane). For the computation, we consider the longitudinal bed slope S=0.05, sediment concentration at the edge of the driving layer $c_{h}=0.01$, Rouse number Z=0.4 and erosion coefficient $N=5\times 10^{-6}$. The colour bar shows the growth rate of perturbations. The white line characterizes the neutral stability curve (Ω=0). The left and right sides of the white line represent the unstable (Ω>0) and stable (Ω<0) zones, respectively. From the numerical experiment, it is found that the neutral stability curve on the G-k plane does not vary significantly with S, $c_{h}$, Z and N.

### (c) Perturbation fields

**Figure 13.**
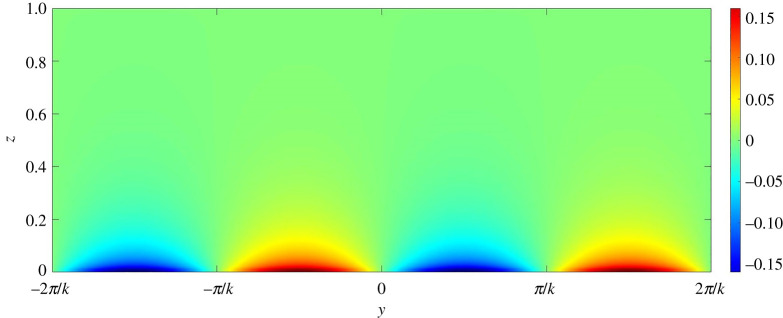
Longitudinal velocity perturbation field on yz plane. (Online version in colour.)

**Figure 14.**
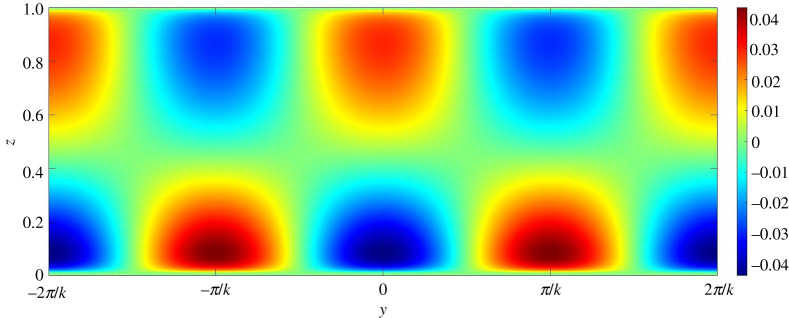
Lateral velocity perturbation field on yz plane. (Online version in colour.)

**Figure 15.**
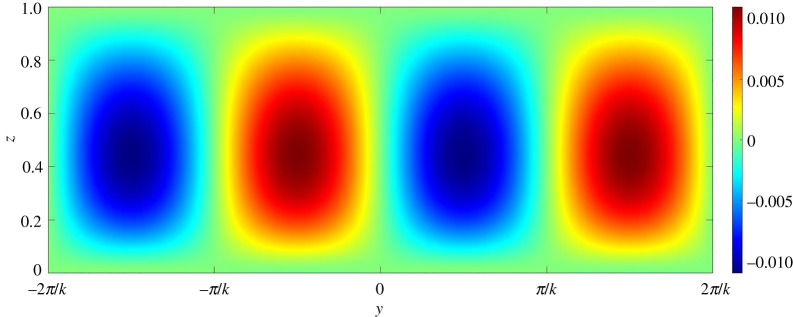
Vertical velocity perturbation field on yz plane. (Online version in colour.)

**Figure 16.**
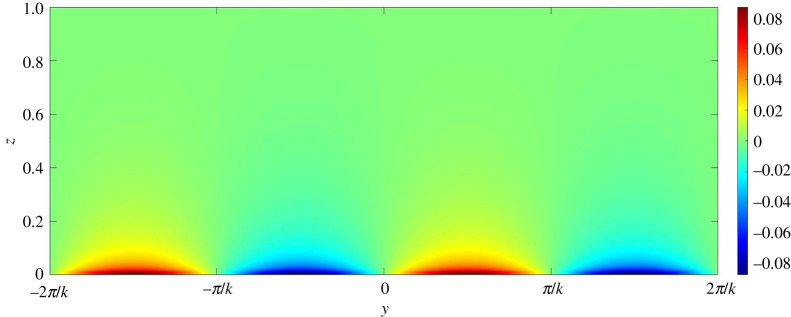
Sediment concentration perturbation field on yz plane. (Online version in colour.)

**Figure 17.**
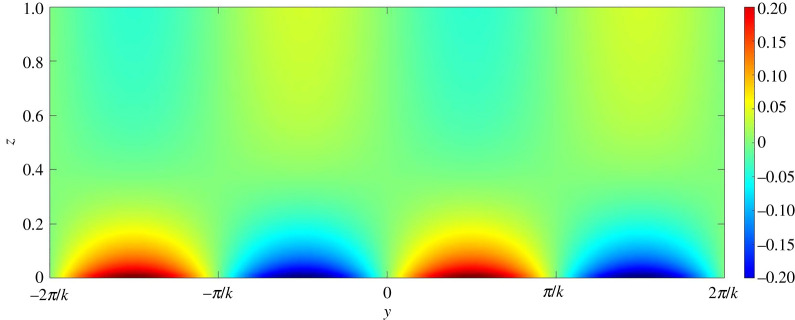
Pressure perturbation field on yz plane. (Online version in colour.)

To gain further insights into the mechanism of channel formation, it is necessary to explore the perturbation fields. In this regard, figures 13–17 present the perturbation fields of the longitudinal velocity, lateral velocity, vertical velocity, sediment concentration and pressure, respectively. The dimensionless wavenumber is set as unity. In figures 13–17, the gravitational parameter G=40, longitudinal bed slope S=0.05, sediment concentration at the edge of the driving layer $c_{h}=0.01$, Rouse number Z=0.4 and erosion coefficient $N=5\times 10^{-6}$ are considered. The perturbation fields of longitudinal velocity and concentration vary significantly in the near-bed flow region (figures 13 and 16), indicating that the far-bed flow does not influence the instability process. This observation reinforces the fundamental assumption, which states that the driving layer of the turbidity currents contributes solely to the instability process. The alternate positive and negative concentration perturbations characterize the trough and the crest of a developing submarine channel, respectively (figure 16). The positive concentration perturbation causes erosion, whereas the negative concentration perturbation yields deposition. A reduction in the suspended sediment concentration results in a decrease in the hydrostatic pressure and vice versa (figure 17). Consequently, a pressure gradient is generated in the lateral direction. The developed pressure gradient drives the flow from the trough to the crest, manifesting the counter-rotating longitudinal vortices (figures 14 and 15). The longitudinal vortices cause the flow at the crest to accelerate and to decelerate at the trough (figure 13).

It is important to mention that the perturbations of the longitudinal velocity, lateral velocity, vertical velocity and sediment concentration vanish at the edge of the driving layer due to the associated boundary conditions. To test the sensitivity of the perturbation fields to the boundary conditions, we perform the numerical experiments assuming the vanishing gradient of the perturbations of the longitudinal velocity, lateral velocity, vertical velocity and sediment concentration at the edge of the driving layer. Note that this consideration allows the perturbations to have finite values in the driven layer. We observe that the qualitative nature of the instability mechanism becomes almost insensitive to the change in boundary conditions at the edge of the driving layer.

The flow in fluvial and marine environments is driven by different mechanisms. The basic difference is that in turbidity currents, the gravity acts on the suspended particles, whereas in fluvial sediment transport, the gravity acts on the fluid. However, the longitudinal features in both the fluvial and marine environments are triggered by the instability process. Therefore, we try to link the present observations with the seminal work of Colombini [44], who examined the development of sand ridges in a fluvial environment. He found that the counter-rotating longitudinal vortices play a destabilizing role. Similarly, this study reveals the appearance of counter-rotating longitudinal vortices (figure 14), which reinforce the instability process.

## 5. Conclusion

We explore the submarine channel formation driven by turbidity currents interacting with an erodible bed from the perspective of the linear stability analysis. The analysis stands on the three-dimensional continuity and momentum equations of flow, advection–diffusion equation of suspended sediment concentration and Exner equation of bed evolution. The flow model within the driving layer considers a parabolic profile for the turbulent diffusivity, which yields a good matching of the computed profiles of base velocity and suspended sediment concentration with the experimental data. The instability process depends on several key parameters: gravitational parameter, longitudinal bed slope, sediment concentration at the edge of the driving layer, Rouse number and erosion coefficient. An increase in gravitational parameter, longitudinal bed slope, concentration at the edge of the driving layer and erosion coefficient plays a destabilizing role, whereas an increase in Rouse number plays a stabilizing role.

For a given set of pertinent parameters, the instability mechanism characterizes the maximum growth rate for a given critical lateral wavenumber. The critical wavenumber reduces with an increase in gravitational parameter, longitudinal bed slope, sediment concentration at the edge of the driving layer and erosion coefficient. However, it increases with the Rouse number. The instability process favours the development of a plane bed when the gravitational parameter, longitudinal bed slope, concentration at the edge of the driving layer and erosion coefficient exceed their upper threshold values, and the Rouse number remains below its lower threshold value.

The present formulation provides an enhanced understanding of the submarine channel formation driven by turbidity currents. However, it stands on a few assumptions. The analysis considers the Boussinesq approximation, wherein the particle loading yields a trivial density variation. For a moderate sediment concentration, the interaction between the particles alters the turbulent diffusivity and the terminal fall velocity [45]. In addition, this study presumes a linear relation between the entrainment flux and the bed shear stress. The entrainment flux is more likely to follow a nonlinear function [46]. Despite some approximations, the present formulation provides an insight into the description and the nature of the instability mechanism. However, there remains scope to further extend the present model by considering the moderate sediment concentration and the nonlinear relation between the entrainment flux and the bed shear stress.

## Data Availability

The numerical code is accessible from the following link: https://docs.google.com/document/d/1iBDLob9c-3L4fPXFt72Q6D9v1eNghEi98KWT6uEXC70/edit?usp=sharing.
